# The levels and trends of contraceptive use before first birth in India (2015–16): a cross-sectional analysis

**DOI:** 10.1186/s12889-020-08917-w

**Published:** 2020-05-24

**Authors:** Pragya Singh, Kaushalendra Kumar Singh, Anjali Singh, Anjali Pandey

**Affiliations:** 1 Department of Statistics, Banaras Hindu University, Varanasi, Uttar Pradesh 221005 India; 2 Department of Statistics,Amity Institute of Applied Sciences, Noida, Uttar Pradesh 201303 India

**Keywords:** Contraceptive, Ever-married, First birth, Logistic regression, Odds ratio

## Abstract

**Background:**

Indian women are more prone to first birth at a relatively younger age after marriage. Also, we do not have sufficient literature available that focuses on contraceptive use before first birth. The analysis of the present study was done using data from the fourth round of National Family Health Survey (2015–16), India. The objectives of the present study were to measure the levels and trends of contraceptive use before first birth among Indian ever married women, aged 15–34 years.

**Methods:**

The study includes 279,896 ever married women aged 15–34 years at the time of the NFHS-4 survey. To identify the socio-demographic determinants governing the pioneering study behavior, multivariable techniques have been used in the analysis. The statistical significance of the relationship between socio-demographic factors and contraceptive use prior to first birth was tested using a chi-squared test for association. Hosmer Lemeshow statistics and Nagelkerke R square have been used to check how well the logistic regression model fits the data. Map of India showing different zonal classification is made using the ArcGIS software version 10.3.

**Result:**

The trends of contraceptive usage show a decline in use before first birth and the various socio-demographic factors affecting the use of contraceptive before first birth are religion, caste, education, wealth index, media exposure, age at marriage and the zonal classifications.

**Conclusion:**

The noticeable result in this study is the comparative decline in contraceptive use by women in India before first birth in NFHS-4 with respect to previous NFHS done in India. The likelihood of using contraception before first birth is significantly affected by factors like place of residence, religion, caste, current age of women, age at marriage, education level of women, wealth index, media exposure and zonal classification.

## Background

Worldwide, the unmet need for family planning is highest among women who are younger than 20 years of age, and lowest among women aged 35 years and older; these differences are widest in South Central Asia, including India [1, 2]. In India, women give birth to their first child at a relatively young age. The median age at first birth in India is 21 years for women aged 25–49 years [1]. Relatively low age at first birth is still persisting despite the reduction in fertility rate and increase in age at marriage as well as educational status. As per NFHS-4, 26.8% of women are married before 18 years of age while this was 47.4% in NFHS-3. This data clearly shows a decreasing trend in age at marriage but still a larger section is married before 18 years.

It is observed that the percentage use of contraceptives has declined, which is a serious concern for healthcare because we are still a victim of population growth as well as maternal deaths. Maternal deaths are projected to be 1.8 times higher in women without contraceptive use [3]. Among many interventions, contraceptive use to prevent unwanted pregnancies is one of the most cost-effective ways of reducing maternal deaths [4]. The less use of contraception and still having an unmet need in the population will increase the unwanted pregnancy which is directly going to increase the abortion in society. Greater contraceptive use allows births to be spaced better and reduces the chances of accidental pregnancies and population rise.

Family Planning 2020 is an initiative to expand contraceptive use to 120 million additional women and girls by 2020 [5]. India has continued its efforts to expand the range and reach of contraceptive options through rolling out new contraceptives and delivering a full range of family planning services at all levels. A recent analysis of DHS data from 52 developing countries found that a large proportion of women cite fear of contraceptive side effects and infrequent sex as reasons for not using contraception [6].

Meeting the unmet need for contraception to delay first birth is vital for several reasons. Above all, it enables countries to respect the reproductive rights of young women and protect them from early and risky pregnancies [7]. It also provides an opportunity to promote population stabilization by delaying first birth and thus increasing the spacing between generations [8]. There is not much literature available regarding the demand for contraception to delay first birth and the hindrances that prevent married young women who wish to postpone their first pregnancy.

In India, cultural norms force young people to prove their fertility as soon as possible after marriage, and that promoting birth spacing until after the first birth is futile [9–11]. As a result of this, neither family elders nor health care providers attempt to facilitate contraceptive use among the young couple especially before first birth. The other possible obstacles discouraging use of contraception before first birth are young women’s have lack of knowledge of how pregnancy occurs and about the contraception and where its supplies are available; limited access to sex education in the school and home; limited mobility and freedom to access clinics and contraceptives; unequal power relations within marriage and the experience or fear of spousal violence that can inhibit young women from acting on their desire to space births; the cost of supplies and of reaching supply outlets; and barriers relating to quality of care, notably the attitude of providers [12]. Early pregnancies can cause a serious threat to women’s health conditions [13]. Thus, delaying the risk of first pregnancy in early marriage is desired for the health of the mother as well as a child because one of the main factors responsible for morbidity and mortality among women in a reproductive age group are the complications associated with pregnancy and childbirth. It has also been studied how various socio-demographic characteristics of the households in which the woman belongs affects the behavior of women to use the contraceptive.

The purpose of the current study is to determine whether changes have occurred in the prevalence of contraceptive use before first birth in India during the past decade and to focus on levels and trends of use before first birth for ever-married women aged 15–34 years of India.

## Methods

### Study design and data source

For the present study and analysis, data is derived from a large scale survey named National Family Health Survey (NFHS) conducted by the International Institute for Population Sciences (IIPS), Mumbai with the funding of ORC Macro and Bill & Melinda Gates Foundation. NFHS is the multi-round survey started in the year 1992–93 and continuously performed at every 5 year interval except the last round of the survey, completed in the year 2015–16. Till now four rounds of surveys have been conducted starting from NFHS-1 (1992–93), NFHS-2 (1998–99), NFHS-3 (2005–06) and NFHS-4 (2015–16) by IIPS under the supervision of Ministry of Health and Family Welfare, Government of India. It gathered information from women and men who were in their reproductive age groups 15–49 years and 15–54 years respectively. As per the objective survey provides estimates of fertility, mortality, maternal and child health, family planning practices and reproductive health, HIV/AIDS and awareness, nutritional status, utilization and quality of health and family planning services across 29 states/union territories and India. It is specially designed to measure the utility and success of family planning services among the Indian population. The detailed description of the study design, sampling procedure, frame, and non response rate are published in the round specific reports (IIPS, 1995; 2018). In total, 699,686 women were successfully interviewed (aged 15–49 years) in NFHS-4 and in the present analysis, we have included 279,896 ever-married women aged 15–34 years at the time of the survey. To compare the changing behavior in the use of contraception before first birth we have made use of NFHS-3, NFHS-2 and NFHS-1 data.

### Description of study variable and measurements

#### Dependent variable

The outcome variable of this study was contraceptive use which we have defined in such a way that it has two categories: It assigns a value 1 for the use of any form of contraceptive method to prevent or delay their first birth and 0 to all those who either never used any contraceptive method or used it after having their first birth. Data on contraceptive use was obtained through the women’s questionnaire.

#### The explanatory variables used in this study are

The background variables selected in the study are socio-economic variables i.e. place of residence, religion, caste, education, wealth index, current age group, age at marriage, media exposure and zonal classification. The place of residence of the respondent is classified into two groups’ urban and rural. Religion consists of three groups namely Hindu, Muslim and others. The others group consisted of Christians, Sikhs, Buddhist/Neo-Buddhist, Jain, Jewish, Parsi/Zoroastrian Donyi polo and others. Caste was categorized into three classes namely Scheduled Caste and Scheduled Tribe (SC/ST), Other Backward Classes (OBC) and others. The educational qualification of women was classified into four categories namely no education, primary, secondary and higher. Wealth index was grouped into three categories namely rich, middle and poor. The current age groups considered in our study were stratified into four age groups as 15–19, 20–24, 25–29, and 30–34 years age of women at the time of the survey. The age at marriage of the respondents was classified into three groups as ≤16, 17–22 and ≥ 23 years. Exposure to media variable was created using the information from three questions from the questionnaire: Do you read a newspaper or magazine almost every day, at least once a week, less than once a week or not at all?, Do you listen to the radio almost every day, at least once a week, less than once a week or not at all? and do you watch television almost every day, at least once a week, less than once a week or not at all?. The final variable exposure to media was then recoded into the following three categories (i) not at all (ii) weekly or less than weekly and (iii) daily exposure. The final variable exposure to media was then recoded into the following three categories (i) not at all, if the answer to all the above three questions were “not at all”, (ii) weekly or less than weekly if at least any one of the answers being “at least once a week/less than once a week” and, (iii) daily exposure, if the response to any one of the above three questions is “every-day”. The twenty-nine states were stratified into four zones: North, South, East, and West. The states included in these zones are given in Table 1.

**Table 1:** Classification of states and union territories under different zones

Zonal Classification	States Union territories included
North	Uttar Pradesh, Bihar, Chhattisgarh, Madhya Pradesh, Rajasthan, Delhi, Haryana, Uttaranchal, Punjab, Himachal Pradesh, Jammu & Kashmir
South	Andhra Pradesh, Karnataka, Kerala, Tamil Nadu, Telangana
East	Orissa, West Bengal, Jharkhand, Tripura, Meghalaya, Assam, Sikkim, Arunachal Pradesh, Nagaland, Manipur, Mizoram
West	Gujarat, Maharashtra, Goa

#### Data management and analysis

After data cleaning and recoding some of the variables to suit the objective of this study, descriptive statistics was prepared to summarize the data. To identify the socio-demographic determinants governing the pioneering study behavior, multivariable techniques have also been used in the analysis. For the multivariate analysis, a statistical technique used in this study is logistic regression. Since the dependent variable used in this study contraceptive use is a dichotomous variable, logistic regression technique is employed to assess the net influences of multiple explanatory variables on the use of contraceptive use prior to first birth after controlling other relevant predictor variables. Hosmer Lemeshow statistics and Nagelkerke R square have been used to check how well the logistic regression model fits the data. Crude and adjusted odds ratios and their 95% confidence intervals (CI) were estimated. Cross-tabulation with frequencies and percentage of each variable was performed. Moreover, the statistical significance of the relationship between socio-demographic factors and contraceptive use prior to first birth was tested using a chi-squared test for association. Map showing the percentage use of contraceptives in different states and their zonal classification is made using ArcGIS version 10.3 software. The analysis is done by STATA software.

## Results

### Trends in contraceptive use before first birth in India: 1992–2016

**Fig. 1:**
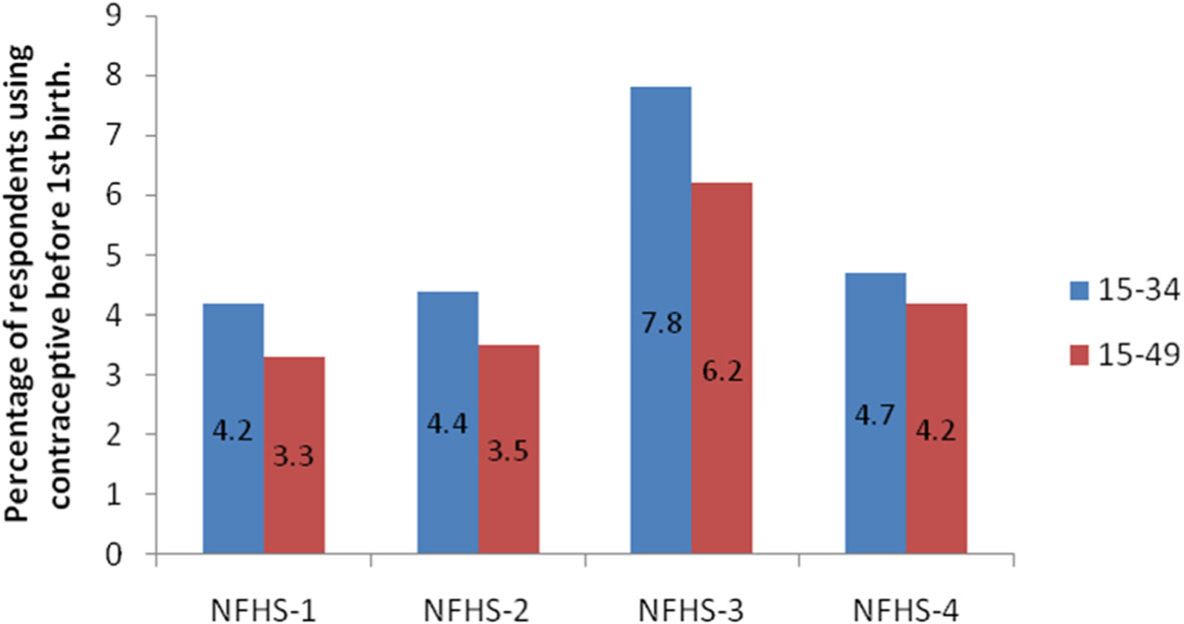
The difference in percentage of contraceptive use before first birth in all 4 rounds of NFHS survey

Figure 1 shows the contraceptive use pattern before first birth for the four surveys and the percentage point differences for four time periods: 2016–2005, 2005–1999, 1999–92, and 2016–1992. A continuous increase can be seen from NFHS-1 to NFHS-3 but thereafter a sharp decline can be noticed. It has been dropped to 4.7 and 4.2% in NFHS-4 as compared to 7.8 and 6.2% in NFHS − 3 for ages 15–34 and 15–49 years respectively.

**Fig. 2:**
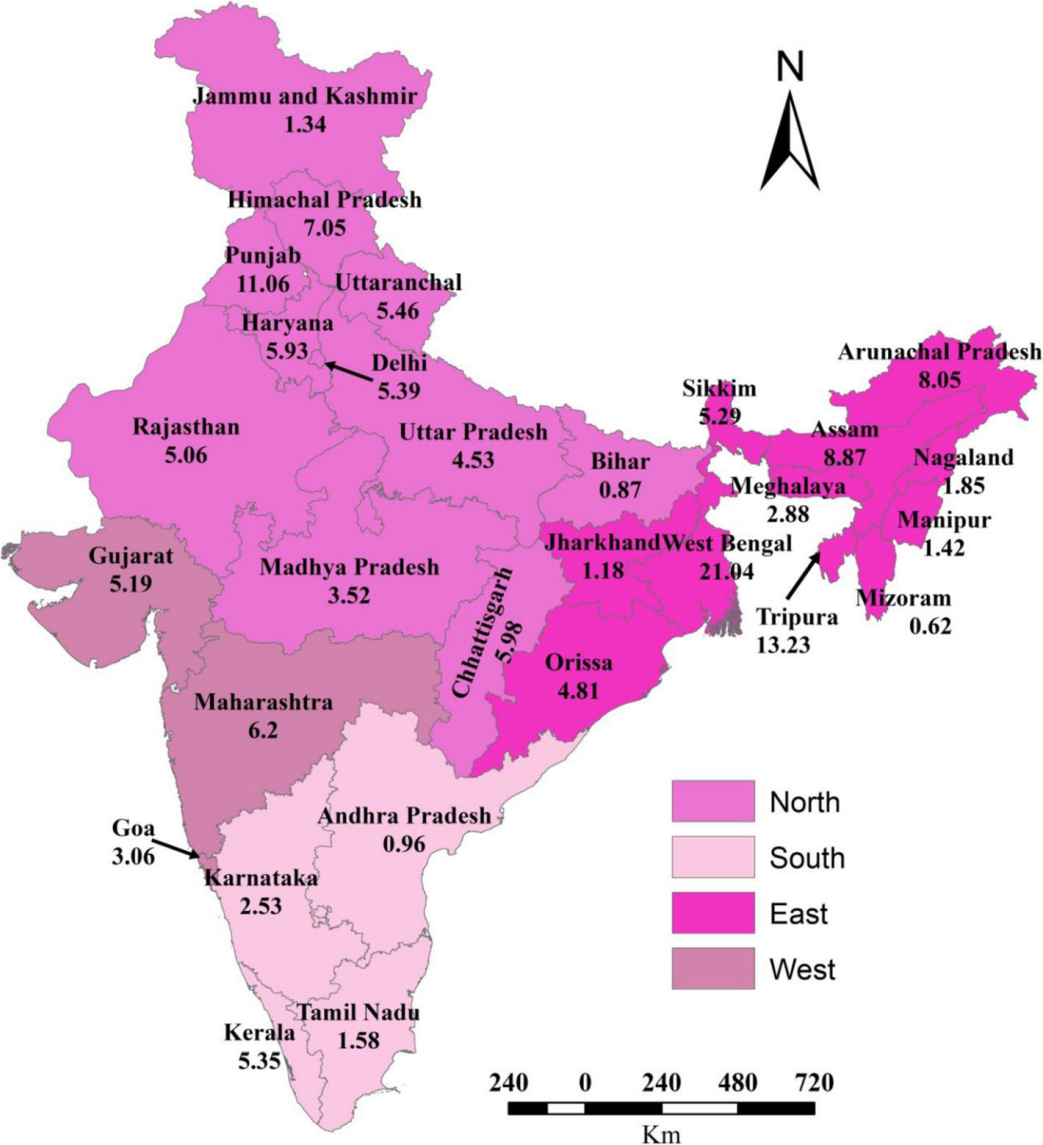
Map of India showing the zonal classification and percentage use of contraceptive before first birth across the states of India,2015–16. Source: Authors generated the map using ArcGIS version 10. *Scale of the map: 1 cm on paper = 170 km on ground

The map shows the zone wise classification described in Table 1 of various states/union territories and also their percentage use of contraceptives before first birth. From the map, it is visible that in Northern India: Punjab (11.06%) and Himachal Pradesh (7.05%) are the highest users of contraceptive before first birth while in the South: Kerala (5.35%) is the leading state with women using contraceptive before first birth. In the East: West Bengal (21.04%) while in the West: Maharashtra (6.09%) is the leading state in using contraceptives before first birth (Fig. 2).

Table 2 describes the socio-demographic characteristics of the respondents considered in the study. Majority of the respondents are residents of rural areas. In the study are around 74% of women residing in rural areas and 26% of them residing in urban areas. Maximum proportions of women are Hindus (76.3%) followed by 13.3% Muslims and 10.3% are of other religions. Around 37% of women belong to scheduled caste and scheduled tribes, 42% to other backward classes and the remaining 21% to other castes. Almost one fourth (25%) of the women have no formal education, 14% have attained primary education, almost half of them (50%) have reached secondary education and only 11% have higher education. Women belonging to poor, middle and rich wealth index are 43, 21 and 36% respectively. The current age structure of the ever-married women has been studied which consists of 5.9% of women belonging to the age group 15–19 years while 27.3, 35.1, and 31.6% of women belong to age group 20–24, 25–29 and 30–34 years respectively. From the tabulation of the variable labeled as age at marriage, it was observed that 30% of the women were married at ages less than equal to 16 years, slightly more than half 52.2% women got married between ages 16 to 22 years and 17.59% women were married when their age was 23 years or more. A large proportion of women are exposed to media regularly. More than half (57%) of women had exposure to media almost every day, 21.9% had no exposure to any kind of media and 20.7% had weekly or less than weekly exposure. Region-wise distribution of women depicts that 55.46, 11.36% of women are from the northern and southern region and 25.26, 7.92% from the eastern and western region.

**Table 2:** Percentage distribution of respondents according to different socio-demographic variable

Socio demographic variable	Percentage distribution	Percentage of women using contraception before firth birth
**Place of Residence**		
Urban	26.21	5.34
Rural	73.79	4.53
**Religion**		
Hindu	76.31	4.77
Muslim	13.31	4.37
Others	10.38	5.02
**Caste**		
SC/ST	37.41	4.57
OBC	41.98	4.01
Others	20.61	6.54
**Educational Qualification**		
No education	25.16	3.11
Primary	14.17	4.34
Secondary	49.84	5.13
Higher	10.83	7.26
**Wealth Index**		
Poor	43.27	3.77
Middle	21.18	4.75
Rich	35.55	5.93
**Current age group**		
15–19	5.99	2.79
20–24	27.26	5.25
25–29	35.12	5.05
30–34	31.63	4.34
**Age at marriage**		
< =16	30.14	4.29
17–22	52.27	4.81
23–34	17.59	5.31
**Media Exposure**		
Not at all	21.95	3.09
Weekly/less than weekly	20.73	4.46
Everyday	57.32	5.48
**Zones**		
North	55.46	4.33
South	11.36	2.22
East	25.26	6.47
West	7.92	5.73
**Total**	**100**	**4.74**
**N**	**279,896**	**13,273**

Table 2 also presents the percentage distribution of respondents using contraceptive before first birth. It is higher for women whose place of residence was urban (5.3%) as compared to those residing in rural areas (4.5%). This percentage is lower for women belonging to Muslim (4.3%) and Hindu (4.7%) religious groups as compared to other religious groups (5%). Analyzing the caste wise variation the percent of women trying to postpone first birth is highest for other caste groups (6.5%) and almost equal for SC/ST and OBC category. A positive relationship is observed between the education status of women and the use of contraceptives before first birth. The percentage increases with the increase in educational level from 3.1% percent for no education to 7.3% with a higher educational group. Further, with an increase in the wealth index, the percent use also increases. It is maximum for women belonging to a rich household (5.9%) and a minimum for the women belonging to a poor household (3.7%). The maximum use of contraceptives is in the women age group 20–24 years (5.3%) and minimum in the women age group 15–19 years (2.8%). While with the increase in age at marriage, the percentage of women portraying the study characteristics, vary from 4.3 to 5.3% for age group less than equal to 16 years and 23–34 years respectively. It is noticed that as media exposure is rising the percentage use of contraceptives before first birth is also rising. As for those who are not at all exposed to the media this percentage is 3.1%, for those with weekly or less weekly it is 4.5% while 5.5% of respondents with daily exposure. Geographical variations were marked as the eastern zone has the highest percentage of women (6.5%) depicting this study characteristic and southern zone having the least (2.2%).

Table 3 presents the result of regression analysis. To study the impact of statistically significant predictor variables on a dichotomous dependent variable logistic regression analysis is performed. The prediction accuracy for our model is 92.3% and the *p*-value for Hosmer Lemeshow statistics is 0.849 (>.05) implying that our logistic model fits the data quite well. The value of Nagelkerke R square (pseudo R square) is 0.182 suggesting that our model is a strong improvement over a null model with no predictors. Having a view on the explanatory variable place of residence we find that women residing in urban areas were significantly more likely to use the contraceptive method before first birth as compared to those residing in rural areas and the difference is highly significant. Women in urban areas had 1.18 (95% CI: 1.4–1.2) times higher odds as compared to women belonging in rural areas. However, the result was insignificant when we shifted from crude to an adjusted odds ratio. The adjusted odds for Muslims and other religious groups were found to be 0.93 (95%CI: 0.89–0.99) and 0.76 (95%CI: 0.71–0.81) respectively taking Hindus as the reference category and it is statistically significant. The contraceptive prevalence rate before first birth is highest for other category women considering the SC/ST group as the reference category. The crude odds ratio for others is 1.45 (95%CI: 1.39–1.52) and adjusted odds are 1.20 (95%CI: 1.14–1.26) which means that contraceptive use among the women belonging to other caste group is 1.45 and 1.20 times higher compared to SC/ST women. A significant increase in odds is noticed as we move from women with no education to women with higher education who have ever used contraceptives before first birth. Women with higher education, which means women with higher than secondary education have 2.43 (95%CI: 2.29–2.59) times higher odds as compared to women with no education. However, when the result is controlled considering all other explanatory variables the adjusted odds are still significant and high for women with higher education but lower than the crude odds ratio. The adjusted odds for education are 1.2 (95%CI: 1.13–1.29), 1.3 (95%CI: 1.24–1.38) and 1.8 (95%CI: 1.70–1.97) times higher for women having primary, secondary and higher education as compared to no education taken as a reference category. Another important variable is the socioeconomic variable in this study which is represented by wealth index. Having a glance at crude and adjusted odds for different categories of wealth index created we find that a significantly higher proportion of women are using contraceptives prior to first birth who are rich in comparison to their counterparts. The respondents belonging to households with rich wealth index have 1.61 (95%CI: 1.54–1.67) times higher odds than women belonging to households with poor wealth index. Similarly, for adjusted odds, it is 1.37 (95%CI, 1.29–1.44) times higher as compared to the poor wealth index taken as the reference group. The odds for the age at marriage group 17–22 years are 1.13 (95%CI: 1.08–1.17) which is significantly higher than the age at marriage group <=16 years which is considered as reference category, the odd for 22–34 years age at marriage group is highest and is 1.25 (95%CI: 1.18–1.31). The odds for married women using contraceptives before first birth and exposed to the media every day is higher than those who are exposed weekly or less weekly. The odds for those who are exposed to media every day are 1.82 (95%CI: 1.72–1.91) which is significantly higher than those who are not exposed to any media exposure at all. The odds for adopting any contraceptive methods to delay first birth is 0.50 (95%CI: 0.46–0.54) for southern region which goes up to 1.34 (95%CI: 1.26–1.42) and 1.52 (95%CI: 1.47–1.58) in western and eastern region pointing towards rise in chances of depicting the study characteristic than the women who live in northern zone. With all the other predominant variables being controlled, the different zones are offering ineludible variations.

**Table 3:** Logistic regression result of the contraceptive use to delay first birth among ever-married women (aged 15–34 years in India, 2015–16)

Socio Demographic Variable	Crude odds ratio		Adjusted odds ratio	
	Odds ratio	C.I(95%) Lower Upper	Odds ratio	C.I(95%) Lower Upper
**Place of Residence**				
Rural	Ref	–	Ref	–
Urban	1.18**	1.14 1.23	0.92	0.88 0.96
**Religion**				
Hindu	Ref	–	Ref	–
Muslim	0.91**	0.86 0.96	0.93*	0.88 0.99
Others	1.05	0.99 1.11	0.76**	0.71 0.81
**Caste**				
SC/ST	ref	–	Ref	–
OBC	0.87**	0.83 0.90	0.88**	0.84 0.92
Others	1.45**	1.39 1.52	1.20**	1.14 1.26
**Educational Qualification**				
No education	Ref	–	Ref	–
Primary	1.41**	1.32 1.50	1.21**	1.13 1.29
Secondary	1.68**	1.60 1.76	1.31**	1.24 1.38
Higher	2.43**	2.29 2.59	1.83**	1.70 1.97
**Wealth index**				
Poor	Ref		Ref	
Middle	1.27**	1.21 1.33	1.13**	1.07 1.19
Rich	1.60**	1.54 1.67	1.36**	1.29 1.44
**Current Age group**				
15–19	Ref	–	Ref	–
20–24	1.93**	1.75 2.12	1.90**	1.72 2.09
25–29	1.85**	1.68 2.04	1.83**	1.66 2.01
30–34	1.58**	1.43 1.74	1.57**	1.42 1.74
**Age at marriage**				
< =16	Ref	–	Ref	–
17–22	1.12**	1.08 1.17	0.93**	0.89 0.97
> =23	1.25**	1.18 1.31	0.84**	0.79 0.89
**Media**				
Not at all	Ref	–	Ref	–
Weekly and less weekly	1.46**	1.37 1.55	1.25**	1.17 1.33
Everyday	1.81**	1.72 1.91	1.43**	1.34 1.52
**Zones**				
North	Ref	–		–
South	0.50**	0.46 0.54	0.43**	0.40 0.47
East	1.52**	1.47 1.58	1.70**	1.63 1.77
West	1.34**	1.26 1.42	1.19**	1.11 1.26

** *p* < 0.01, *p < 0.05

## Discussion

Despite, the awareness regarding contraceptives has increased [14, 15] our results surprise us with the decline in the percentage of contraceptive use before first birth (Fig. 1) which is a matter of concern. One of the possible reasons for the decline is because women are getting married late which prevents the delay of first birth after marriage. NFHS-4 reports clearly show an increase in age at marriage of Indian women [1].

In the present analysis, women residing in urban areas have higher odds and were significantly more likely to use the contraceptive method before first birth as compared to those residing in rural areas and the difference is highly significant (Table 3). Similar results were reported in some previous studies [12, 16]. In this study, we find there are certain religious groups like Muslims and others who use fewer contraceptives prior to first birth as compared to the Hindu religious group. There are certain social norms in different religions that affect the use of the contraceptive method before or after the first pregnancy [16, 17]. This result is in consensus to many other such findings.

Female education, particularly completion of primary school and secondary school, has emerged as strongly related to lowered fertility [16]. As many studies have shown concerning contraceptive use more generally [14], women’s education and household economic status are associated with the practice of contraception—in this case with contraceptive use among young women reporting a demand for contraception to delay their first pregnancy. A similar result is observed in this study. A significant increase in the odds ratio is noticed as we move from women with no education to women with higher education who have ever used contraceptives before first birth.

In most developing nations, there is a wide gap between the socioeconomic statuses of the poor and rich that interferes with the policies of the family planning program. The odds ratio for rich women is 1.61 times higher than the poor which is statistically significant. A similar result is obtained in many earlier studies [18–20].

Considering the current age composition the maximum use of contraceptives before first birth is in the age group 20–24 years which declines with an increase in age. However, the use of contraceptives prior to first birth in the age group 15–19 years is less as compared to the age group 20–24 years which is one of the high spots of this study. This is a clear indication that there is a decline in contraceptive use before first birth in the younger age group (15–19 years) as compared to the previous survey i.e. NFHS-3 [21]. A similar pattern of variation in the age group can be observed in other studies [16, 22]. An increase in age at marriage is accompanied by an increase in the use of contraceptives prior to the first birth. The odds for age at marriage group 17–22 years are 1.13 which is significantly higher than the age at marriage group <=16 years, the odds for age at marriage group 22–34 years is highest and is 1.25. This gives a clear picture of increasing odds with an increase in age at marriage which states that when women are married late still there is a higher chance that they will use contraceptives before first birth. 8% of women between 15 and 19 years of age were either already mothers or pregnant [1]. NFHS-4 data also reveals that between 2005 and 2006 and 2015–2016, the percentage of women whose current age is 20–24 years, married before 18 years of age dropped by 21%.

An increase in media exposure shows increased use of contraception before first birth. The odds for married women using contraceptives before first birth and exposed to media every day is higher than those who are exposed weekly or less weekly or no media exposure (Table 3). This is similar to a previous study showing that exposure to mass media can be an important means to improve knowledge and initiate women to practice any form of modern contraception before first birth or to increase the spacing between two births [22]. Several other studies have shown that women of developing countries who had exposure to the media family-planning campaigns were more likely to opt for a contraceptive technique than the women without any exposure to mass media [23–26]. Regional patterns generally displayed distinctions consistent with the previous studies [14, 16]. As compared to the north zone we found that women of the south zone are less likely to use contraceptives before first birth. This finding may be attributed to the later age at marriage of young women in southern states as compared to the northern region.

### Strength and limitations

The present study must be considered in light of certain limitations. The chances of under-reporting cannot be ruled out as we are aware that the use of contraceptives is a sensitive and often stigmatized subject in our nation, young women may be reluctant to disclose their contraceptive use status. The analytical sample is restricted to fecund, ever-married (currently or formally) women of age group 15–34 years. This criterion may lead us to suffer from selection bias. Also, we have focused extensively on the use of contraception prior to first birth and not on the type of method used because of the unavailability of information about this aspect in NFHS data. Some other variables that could be used to enrich this kind of study are variables governing women’s autonomy and decision making but due to fewer observations, we have skipped them. One of the main strengths of this study is using all the four rounds (1992–93, 1998–99, 2005–06, and 2015–16) of published NFHS data for comparison. This article canvassed the socio-demographic factors determining the levels and trends in the use of contraceptive methods before first birth as well as evokes the curiosity to evaluate and initiate a discussion to help and investigate the reasons for the recent decline. The use of a nationally representative dataset reveals a true representation of the situation of this sub-group of women in the country. Also the association of socio-demographic variables governing the behavior of contraceptive use among women before first birth adds value to the present research.

## Conclusion

The study examined factors influencing contraceptive use before first birth in Indian women. It was evident that education, wealth index, exposure of media, age at marriage, regional classification, caste and religion reveals a significant key relation with the use of contraception before first birth. Findings from the study also noted a decline in the use of contraception before first birth in the past decade (Fig. 1). This finding may be attributed to the later age at marriage of young women. The success of India’s family planning program to a large extent depends upon the shoulders of policymakers, researchers, users and service providers. So, there is a need to ensure and intensify the existing family planning programs and incorporate a focus on young married women making them realize the hazards associated with early childbearing and equipping them with the skills and resources to delay their first birth if they desire.

## Data Availability

Data are available on request from https://dhsprogram.com/Data/

## Abbreviations

NFHS: National Family Health Survey
OBC: Other backward classes
SC/ST: Schedule caste/Schedule Tribes
